# Should I Stay or Should I Go: Partially Sedentary Populations Can Outperform Fully Dispersing Populations in Response to Climate-Induced Range Shifts

**DOI:** 10.1007/s11538-020-00700-7

**Published:** 2020-01-31

**Authors:** Christina A. Cobbold, Remus Stana

**Affiliations:** 1 School of Mathematics and Statistics, University of Glasgow, University Place, Glasgow, G12 8QW UK; 2 Boyd Orr Centre for Population and Ecosystem Health, University of Glasgow, Glasgow, G12 8QQ UK; 3 School of Mathematics, University of Leeds, Leeds, LS2 9JT UK

**Keywords:** Integrodifference equation, Partially sedentary, Population persistence, Moving-habitat model, Climate change, Geographical range shift

## Abstract

Global mean temperatures have increased by 0.72 $^\circ$C since the 1950s, and climate warming is resulting in geographical shifts in the range limits of many species. Climate velocity is estimated to be 0.42 km/year, and if a species fails to adapt to the new climate, it must track the location of its climatically constrained niche in order to survive. Dispersal has an important role to play in enabling a population to shift is geographical range limits, but many species are partially sedentary, with only a fraction of the population dispersing each year. We ask, can partially sedentary populations keep pace with climate or will such populations be more vulnerable to extinction? Through the development of a moving-habitat integrodifference equation model, we show that, provided climate velocity is not too large, partially sedentary populations can outperform fully dispersing populations in one of two ways: (i) by persisting at climate speeds where a fully dispersing population cannot, and (ii) exhibiting higher population densities. Moreover, we find that positive density-dependent dispersal can further improve the likelihood a population can persist. Our results highlight the positive role that non-dispersers may play in mitigating the effects of overdispersal and facilitating population persistence in a warming world.

## Introduction

Climate warming is occurring across the globe (Easterling et al. 1997; Stocker et al. 2013), with a projected future increase in temperature of 0.2 $^\circ$C per decade over the next 30 years (IPCC 2018). Increased temperatures have impacted ecological systems in numerous ways, from inducing phenological shifts, such as earlier tree bud-burst, frog breeding and arrival of migrant birds, to changes in species abundance and distribution (Parmesan and Yohe 2003). Species’ geographical range limits are often constrained by the local climatic conditions (Parmesan 2006), and warming climates have led to shifts in the range limits of many species (Walther et al. 2002; Parmesan and Yohe 2003). Over the last century, alpine plants in Europe (Grabherr et al. 1994) and beach trees in New Zealand (Wardle and Coleman 1992) have exhibited an upward shift in their elevation range, while over 20 species of European butterfly (Parmesan et al. 1999) and 50 species of British birds (Thomas and Lennon 1999) have shown poleward shifts in their geographical range. In butterflies, over the past 40 years, a range shift as large as 200 km has been observed (Parmesan et al. 1999). The speeds of spatial shifts in surface temperature, referred to as the velocity of climate change, have been predicted to be on average 0.42 km yr$^{-1}$ globally (Loarie et al. 2009). Species have shown three adaptive responses to these changing climatic conditions: (i) move and track the location of their climatically defined habitat, (ii) adapt using phenotypic plasticity to survive in their current geographical range or (iii) evolve new strategies for survival in the current geographical range (Parmesan 2006). We focus our attention on the first of these, as there is a growing body of evidence that range shifts have occurred across many taxa world wide (Walther et al. 2002; Kerr et al. 2015), but we still have an incomplete understanding of what species traits facilitate the tracking of climate-induced range shifts.

Previously, it was proposed that good dispersal ability and high fecundity are desired traits for a species to successfully move its range and track the location of a shifting climate (Harsch et al. 2014). However, for some species only a fraction of the population disperses each generation, such as plant populations with seed banks, which puts constraints on the dispersal ability of the population as a whole. Would these partially sedentary species then be among the first to be threatened with extinction under climate warming if they failed to adapt in other ways? In this paper, we use mathematical modelling to address this question and explore conditions for extinction or survival for these partially dispersing populations.

Among the frameworks used to study the effects of climate-induced geographical range shifts, climate envelope and habitat suitability models have received much attention. Habitat suitability models combine climate predictions and correlative statistical models to predict the future location of populations (Bateman et al. 2013). These approaches provide a useful guide to predict the future geographical ranges of species, but as they omit population processes, they are less well suited to the question of whether a species will be able to reach this new geographical range. To address whether an organism can keep pace with climate-induced range shifts, mechanistic models have been adapted to describe the underlying mechanistic processes of reproduction and dispersal driving a population’s spatial distribution. Harsch et al. (2017) gives an overview of the modelling approaches that have been adopted and finds these approaches fall into three main classes: individual-based models (IBMs), reaction–diffusion equations (RDEs), and integrodifference equations (IDEs), the latter two are perhaps the simplest and so have the virtue that model behaviour can be more readily attributed to biological mechanisms.

The first reaction–diffusion model to describe the impact of shifting geographical ranges on population persistence focussed on competing species, and showed that climate-induced range shifts can facilitate competitive coexistence at range boundaries (Potapov and Lewis 2004). Dispersal has been shown to be a “double-edged sword, with too much or too little leading to population extinction”, and the possibility of climate-induced increases in total population (Berestycki et al. 2009). More recently, the RDE framework has been extended to include the effects of species’ behavioural responses to habitat boundaries, showing that habitat preference can enhance persistence (MacDonald and Lutscher 2018). Bridging the gap between empirical studies and these mathematical models, (Leroux et al. 2013) successfully combined the RDEs with the habitat suitability approach to provide a more predictive tool for assessing the impact of climate-induced range shifts.

While reaction–diffusion models have provided many insights, this approach is not well suited to describe organisms with either temporally distinct growth and dispersal phases or frequent long distance dispersal events. Many temperate insect and plant species have separate growth and dispersal phases and exhibit long distance dispersal, and these taxa include many of the species that have exhibited range shifts in response to warming climates. Integrodifference equations can more accurately model such species, by naturally separating growth and dispersal in its formulation. IDEs also have the additional benefit of allowing for a flexible description of movement through the choice of a dispersal kernel, a flexibility that is harder to achieve using RDEs.

The first IDE study of climate-induced range shifts determined the critical climate speed, below which a population could persist and above this a population would go extinct (Zhou and Kot 2011). An extension of this early model to stage-structured populations demonstrated that if climate shifts were rapid, at a speed of an entire range length per life stage generation-time, then a population with a non-moving life stage would not survive (Harsch et al. 2014). Essentially, the non-moving life stages are left behind, leading to the eventual collapse of the population. The presence of non-moving life stages results in a population where only a fraction disperse, and while large climate shifts result in extinction of these partially sedentary populations, it is not clear what would happen under less severe climate scenarios.

There are many examples of partially sedentary species, where only a fraction of the population disperses. One well-cited example is the house finch in eastern North America studied by Veit and Lewis (1996). Only a fraction of both adult and juvenile birds disperse each year. A number bird species have gone from being completely sedentary species to partially migratory in response to recent climate change (Berthold 2001). A partially sedentary population can also be a consequence of life history strategies that give rise to a population that has a non-moving life stage. Plants have non-moving life stages, as seeds are generally dispersed, but adult plants remaining stationary (Li 2012). Similarly, crustaceans frequently have an adult life stage that is relatively immobile, while the juveniles travel long distances, so when viewed from the perspective of the population as a whole, only a fraction of the population disperses per year (Kanary et al. 2014).

In this paper, we present a general one-dimensional model of a partially sedentary population, in which the geographical range for the population shifts at a constant speed, in line with idealised climate shifts. Outside of the geographical range limits the population is unable to survive. Harsch et al. (2014) refers to this type of model as a ‘moving-habitat model’, and we adopt this terminology here. We derive conditions for the critical climate speed, above which the population cannot persist. We demonstrate how this critical speed depends on the fraction of the population that disperses. Counterintuitively, for some climate speeds, we find that partially sedentary populations can in fact do better than populations where the entire population disperses. The partially sedentary population is able to persist and track changing climate conditions while a fully dispersing population is not, and we discuss the conditions under which this scenario occurs (Sect.3.1). Finally, we also consider density-dependent dispersal and show that positive density-dependent dispersal can further improve the likelihood of persistence for a population experiencing climate-induced range shifts (Sect.3.3).

## Model Formulation

We formulate a moving-habitat IDE model for a partially sedentary population with non-overlapping generations. We let $n_t$ denote the population density in year *t*. During the course of a year, the population first grows, and at the end of the growth period, the non-sedentary fraction of the population disperses, giving rise to the population distribution at the start of the next year. Local population growth is described by *f*(*n*), and in the absence of dispersal, the annual population dynamics are described by the difference equation:

$$\begin{aligned} n_{t+1}=f(n_t). \end{aligned}$$

We assume there is no Allee effect and so $f(n)\le f'(0)n$. For numerical examples, we use the well-studied Beverton–Holt growth function,

1 $$\begin{aligned} f(n)=\frac{Rn}{1+((R-1)/K)n}, \end{aligned}$$

where $f(0)=0$ and $R=f'(0)$ is the intrinsic growth rate of the population at low density and *K* is the population carrying capacity.

We next introduce dispersal into the model, by first considering the case of a stationary habitat and then extending this to the full moving-habitat model. We let *x* denote space and assume the suitable habitat is located at $x\in [-\frac{L}{2},\frac{L}{2}]$; outside of the suitable habitat, the species cannot survive. We assume a fraction *p* of the population disperses and a fraction $1-p$ does not. The stationary habitat IDE model is then given by

2 $$\begin{aligned} n_{t+1}(x)=\underbrace{\int ^{L/2}_{-L/2}pf(n_t(y))k(x-y)\,\mathrm{d}y}_{\text {dispersing fraction}}+\underbrace{(1-p)f(n_t(x))}_{\text {non-dispersing fraction}}, \quad x\in \left[ -\frac{L}{2},\frac{L}{2}\right] . \end{aligned}$$

**Fig. 1:**
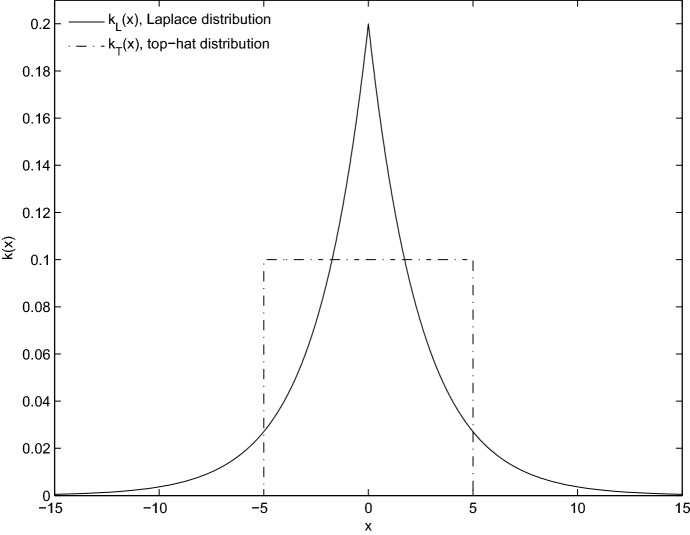
Laplace and top-hat dispersal kernels for a population starting at 0 and dispersing to a location *x* in the domain. As *k*(*x*) is a probability distribution, the area under the curve is 1. The equations for *k*(*x*) are given in Eq. (3), with $\beta =2.5$

At end of the growth phase, $pf(n_t(y))$ individuals disperse from *y* to some location *x* with probability $k(x-y)\mathrm{d}y$, where *k* denotes the dispersal kernel. We assume a symmetric kernel that only depends on the distance travelled during dispersal, with mean dispersal distance $\beta$. In Sect. 3, we focus on two choices of dispersal kernel, the Laplace kernel ($k_L$), which is a good model of both insect and seed dispersal (Taylor 1978; Stevens et al. 2010; Neubert et al. 1995), and the top-hat kernel ($k_T$) whose simplicity allows us to obtain analytical results and can be used to describe plants that disperse via vegetative spread (Duncan et al. 2017). The two dispersal kernels are given by the equations

3 $$\begin{aligned} k_L(x)=\frac{1}{2\beta }e^{-|x|/\beta }\quad \text {and}\quad k_T(x)=\frac{1}{4\beta }\chi _{[-2\beta ,2\beta ]}(x), \end{aligned}$$

where $\chi$ is the indicator function which takes the value 1 inside the set $[-2\beta ,2\beta ]$ and zero outside of this set. Plots of these kernels are given in Fig. 1.

The integral in the first term of Eq. (2) totals all of the dispersing individuals arriving at *x* from locations *y* in the suitable habitat. The second term in Eq. (2) describes those individuals that did not disperse and remained stationary at *x*, so adding these two terms gives the population density at each location *x* at the start of year $t+1$. Finally, we note that we assume that individuals dispersing to locations outside of the suitable habitat will not survive, so Eq. (2) holds for all $x\in [-\frac{L}{2},\frac{L}{2}]$ and $n_{t+1}(x)=0$ for *x* outside of the suitable habitat. We consider two choices for *p*: (i) *p* is a constant, corresponding to density-independent dispersal and (ii) $p=p(n)$, corresponding to density-dependent dispersal. Equation (2) is extensively studied by Lutscher (2008) and Li (2012), and we do not discuss their findings in this section, and instead, we leave the discussion to Sect. 3.3 when we present the results of our moving-habitat IDE.

### Moving-Habitat Model for a Partially Sedentary Population

To introduce the effects of climate warming, we now assume the suitable habitat moves in the positive *x* direction at speed *c*. At time *t*, the suitable habitat is located at $[-\frac{L}{2}+ct,\frac{L}{2}+ct]$. This situation corresponds to a climate-induced habitat shift as observed along a latitudinal or elevational gradient (Zhou and Kot 2011). Our moving-habitat IDE for a partially sedentary population is then given by

4 $$\begin{aligned}&n_{t+1}(x)=\underbrace{\int ^{L/2+ct}_{-L/2+ct}pf(n_t(y))k(x-y)\,\mathrm{d}y}_{\text {\tiny dispersing fraction}}+\underbrace{(1-p)f(n_t(x))}_{{\mathop {\text {\tiny fraction}}\limits ^{\text {\tiny {non-dispersing}}}}},\nonumber \\&\quad \; x\in \left[ -\frac{L}{2}+c(t+1),\frac{L}{2}+c(t+1)\right] . \end{aligned}$$

The limits on the integral term now reflect the moving location of the suitable habitat in year *t*. At the start of year $t+1$, the suitable habitat is located at $[-\frac{L}{2}+c(t+1),\frac{L}{2}+c(t+1)]$, so any individuals dispersing in year *t* to locations *x* outside of this region will die by the start of year $t+1$, similarly, any non-dispersing individuals that were located in $[-\frac{L}{2}+ct,\frac{L}{2}+ct]$ in year *t*, but are not in $[-\frac{L}{2}+c(t+1),\frac{L}{2}+c(t+1)]$ will die by the start of year $t+1$, and hence, Eq. (4) holds for $x\in [-\frac{L}{2}+c(t+1),\frac{L}{2}+c(t+1)]$ and $n_{t+1}(x)=0$ for *x* outside of this domain. *All individuals crossing the habitat boundary are lost.* In the special case of $p=1$, Eq. (4) is the equation studied by Zhou and Kot (2011), and we discuss their work in Sect. 3.

Unless otherwise stated, in all examples and numerical simulations, we use the Beverton–Holt growth function with $K=100$ and a Laplace dispersal kernel with mean dispersal distance, $\beta =2.5$. The length of the suitable habitat patch is $L=1$. Equation (4) is solved in MATLAB using the trapezoidal rule with subintervals of length $\Delta y=L/10000$, yielding a high resolution of the moving-habitat. To enable simulation of the large spatial domains, that are required to track the moving-habitat over a large number of years, and for a large climate speed *c*, we solve Eq. (4) by changing to moving coordinates, thereby significantly reducing the computation time. We initialise the population at $n_0(x)=10$ for $x\in [-L/2, L/2]$ and 0 elsewhere, and then iterate Eq. (4) for 50 years with $c=0$ (no climate change) to allow the spatial distribution to reach a steady state. We then iterate Eq. (4) for a further 500 years with $c\ne 0$. We found that 500 years was sufficient time to determine whether the population would reach extinction, using an extinction threshold of 0.001. If the maximum value of the population in the domain was below 0.001 after 500 years, the population was considered to be extinct.

## Results

**Fig. 2:**
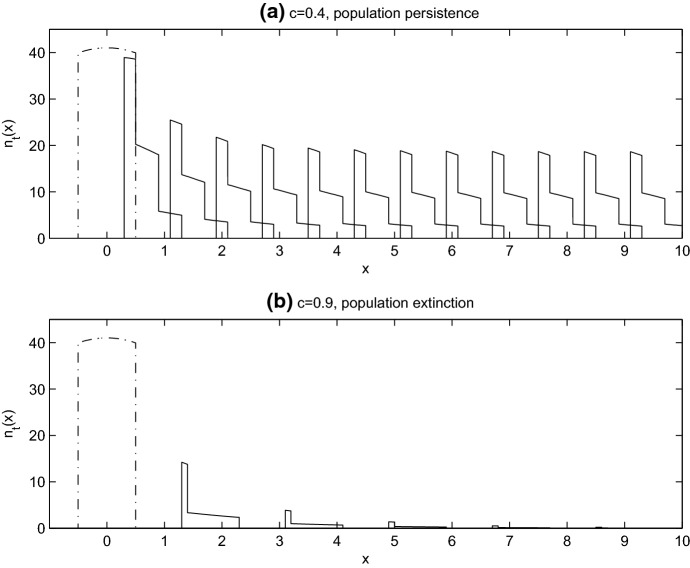
Travelling pulse solutions of Eq. (4). **a**$c=0.4<c^*$, and the population persists. **b**$c=0.9>c^*$, and there is population extinction. In both plots, the dot-dashed line is the initial population distribution. The solid lines are the travelling pulse solution plotted every 2 years. Parameter values are as given in Sect. 2 with $R=6$, $p=0.6$

If we chose parameters $L, R, \beta$ and *p* such that in the absence of climate warming ($c=0$) the population persists, we can then ask the question: Is the population able to persist when $c>0$? Consistent with other moving-habitat IDE and PDE models, we find that there is an upper limit, $c^*$, on the speed of the climate-induced range shift that supports population persistence (Potapov and Lewis 2004; Zhou and Kot 2011). For range shifts below the critical speed, the population can keep pace with the moving-habitat and persist, while speeds above $c^*$ result in population extinction (Fig. 2). In this paper, we focus our results on exploring how the fraction of dispersers (*p*) influences this population persistence condition, $c^*$. In Sect. 3.1, we consider density-independent dispersal, restricting our choice of *p* to a constant, which allows us to conduct a linear stability analysis to establish the conditions for population persistence. In Sect. 3.3, we consider density-dependent dispersal. The dependence of *p* on population density results in a persistence condition that cannot be linearly determined, so in this case, we rely on numerical simulation of the full nonlinear IDE to determine the critical speed $c^*$.

### Density-Independent Dispersal, Constant *p*

Following the same approach as Zhou and Kot (2011, 2013), we obtain an analytical formula for the critical speed, $c^*$, for population persistence. Equation (4) has a travelling pulse solution of the form

5 $$\begin{aligned} n_t(x)=n^*(x-ct) \end{aligned}$$

that moves with a constant speed *c* (see Fig. 2), and the critical speed for persistence is found by studying the stability of these travelling pulse solutions. Substituting (5) into (4) and rewriting (4) using the shifted spatial variables $X=x-ct$ and $Y=y-ct$ gives an equation defining this travelling pulse solution

6 $$\begin{aligned}&n^*(X)=\int ^{L/2}_{-L/2}pf(n^*(Y))k(c+X-Y)\,\mathrm{d}Y+(1-p)f(n^*(X+c)),\nonumber \\&\quad X\in \left[ -\frac{L}{2}+c,\frac{L}{2}+c\right] . \end{aligned}$$

Since $f(0)=0$, we find $n^*(X)\equiv 0$ is a solution of (6). The critical wave speed ($c^*$) is then found by studying the stability of this trivial solution. We let $N_t(x)=n^*(X)+\xi _t(x)$ and linearise (4) about $n^*(X)\equiv 0$ to obtain

7 $$\begin{aligned} \xi _{t+1}(x)={\left\{ \begin{array}{ll} \displaystyle \int ^{L/2}_{-L/2}pR\xi _t(y)k(x-y)\,\mathrm{d}y+(1-p)R\xi _t(x),\\ \quad x\in \left[ -\frac{L}{2}+c(t+1),\frac{L}{2}+c(t+1)\right] ,\\ 0,\quad \text {otherwise}.\\ \end{array}\right. } \end{aligned}$$

Recall, $f'(0)=R$. Making the ansatz, $\xi _t(x)=\lambda ^tu(x-ct)$, Eq. (7) then yields the eigenvalue problem

8 $$\begin{aligned} \lambda u(x)={\left\{ \begin{array}{ll} \displaystyle \int ^{L/2}_{-L/2}pR u(y)k(x-y+c)\,\mathrm{d}y+(1-p)Ru(x+c),\quad x\in \left[ -\frac{L}{2},\frac{L}{2}\right] ,\\ 0,\quad \text {otherwise},\\ \end{array}\right. } \end{aligned}$$

where $\lambda$ is the eigenvalue and *u*(*x*) is the corresponding eigenfunction.

The linear integral operator in (8) is compact, and for positive continuous dispersal kernels such as the Laplace distribution, the operator satisfies Jentzch’s theorem (Horiguchi and Fukui 1996). See also Zhou and Kot (2011, 2013) for a discussion of this theorem. Under these conditions, we are guaranteed the existence of a positive real dominant eigenvalue and corresponding positive eigenfunction. Hence, the trivial steady state is unstable when $\lambda >1$, and when $\lambda =1$, we obtain the stability boundary separating population persistence from extinction.

**Fig. 3:**
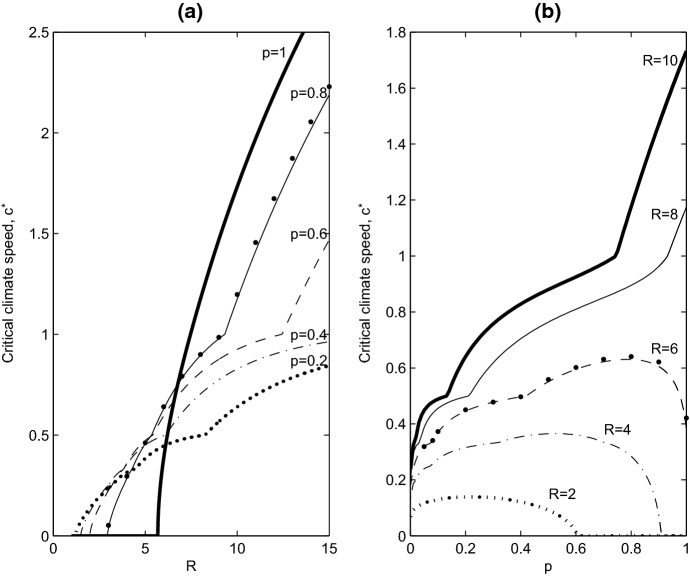
Stability curves calculated from the eigenvalue problem in Eq. (8). **a** Critical wave speed ($c^*$) is plotted as a function of the intrinsic growth rate (*R*) for different dispersing fractions (*p*). **b** Critical wave speed ($c^*$) is plotted as a function of the dispersing fraction (*p*) for different intrinsic growth rates (*R*). Below the curves the population persists, and above the curves the population goes extinct. The large dots in both plots correspond to the stability boundary obtained by solving the full nonlinear IDE directly, demonstrating a close match between the linear analysis and nonlinear problem

Equation (8) is solved numerically using Nyström’s method, as outlined by Zhou and Kot (2013), to find the critical climate speed $c=c^*$ as a function of the other model parameters. Nyström’s method involves discretising the integral in the eigenvalue problem using the trapazoidal rule, and this converts the problem of finding eigenvalues of an integral operator into a problem of finding eigenvalues of a matrix operator which we solve using the *eigs* command in MATLAB. The domain of integration in the eigenvalue problem is $[-L/2, L/2]$, which is divided into $N=2000$ subintervals. The dominant eigenvalue depends on the model parameters, and in particular, it depends on the climate speed, *c*. The critical value of *c*, $c^*$, occurs when the dominant eigenvalue $\lambda =1$. So we find $c^*$ using a root-finding algorithm, and in this case, the bisection method with a tolerance of $10^{-8}$. In Fig. 3a, we illustrate $c^*$ as a function of the intrinsic population growth rate *R*. The dots in Fig. 3a and b are obtained by solving the full nonlinear IDE. We see there is excellent agreement between the linear analysis and the full nonlinear problem.

#### Critical Climate Speed, $c^*$

When the population growth rate is high (e.g. $R>7$), we find that a population in which everyone disperses ($p=1$) performs best, meaning that $c^*_{p=1}>c^*_{p\ne 1}$. The fully dispersing population can persist for higher values of *c* compared to a partially sedentary population. Moreover, as one might intuitively expect, the higher the value of *p* (the dispersing fraction), the larger the value of $c^*$. As suggested by Harsch et al. (2014), for high *R*, a good dispersal ability is indeed beneficial to the population. However, the story changes when we consider lower growth rates. We can now find climate speeds for which the partially sedentary population ($p\ne 0$) persists but the fully dispersing population ($p=1$) cannot, $c^*_{p=1}<c^*_{p\ne 1}$.

In Fig. 3b, we plot $c^*$ as a function of the dispersing fraction *p*. For low to intermediate values of the intrinsic growth rate (*R*), the graph has a maximum located at $0<p<1$. This means that there is an optimum fraction of dispersers, *p*, that gives rise to the largest range of climate speeds (*c*) that allow population persistence. As *R* increases, this optimum fraction of dispersers increases until eventually a fully dispersing population always tolerates a larger range of *c* compared to a partially sedentary population.

**Fig. 4:**
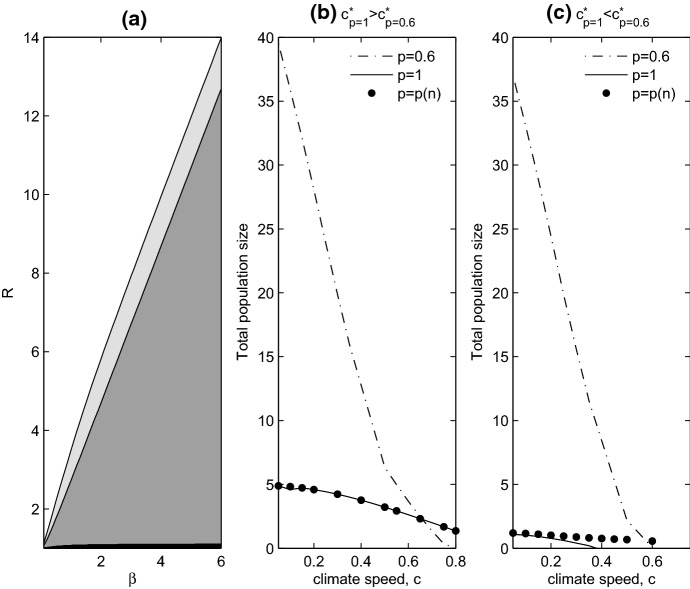
**a** Effects of mean dispersal distance ($\beta$) and intrinsic growth rate (*R*) on persistence and critical wave speeds. In the black region all populations go extinct. In the dark grey region, a fully dispersing population cannot persist, but a partially sedentary population can, and $c^*_{p=1}<c^*_{p_{\ne } 1}$. In the light grey region all populations can persist, but $c^*_{p=1}<c^*_{p_{\ne } 1}$, while in the white region all populations persist, but $c^*_{p=1}>c^*_{p_{\ne } 1}$. In **b** and **c** total population density of the travelling pulse solution is plotted as a function of climate speed. In (b), $R=7.5$ and $\beta =2.5$, corresponding to the scenario where $c^*_{p=1}>c^*_{p_{\ne } 1}$. In **c**, $R=6$ and $\beta =2.5$, corresponding to the scenario where $c^*_{p=1}<c^*_{p_{\ne } 1}$. The dash-dot line indicates the population size of the partially sedentary population ($p=0.6$), the solid line denotes the size of the fully dispersing population ($p=1$), and the large dots correspond to the size of population exhibiting positive density-dependent dispersal, with $a=1$

To make sense of these results, we first note that when $c>L$ the habitat is shifting by more than one habitat length per year. Consequently, non-dispersing individuals will always be left behind by climate change, i.e. will end up outside of the suitable habitat after 1 year. These individuals will therefore not survive between years, and so only dispersing individuals contribute to overall population persistence. Consequently, when $c>L$, decreasing *p* has the effect of lowering the population growth rate of the dispersing fraction, and so a population with $p=1$ will always survive in faster moving climates than one with $p<1$.

When $c<L$ some of the non-dispersing fraction can survive, contributing to the total population density. In this case, $p=1$ may no longer be optimal. Instead, there is a trade-off between the costs of dispersing (overdispersal, in particular, loss due to landing behind the newly available habitat) and costs of not dispersing (loss due to remaining in a section of the old habitat which is no longer suitable). As *c* gets smaller, the cost of dispersing becomes larger and the cost of not dispersing becomes smaller, and so a partially sedentary population ($p\ne 1$) performs better than a fully dispersing population ($p=1$). Increasing the mean dispersal distance, $\beta$, results in an increase in the region of parameter space (light and dark grey regions in Fig. 4a) where partially sedentary populations perform better than fully dispersing populations ($c^*_{p=1}<c^*_{p\ne 1}$), this is because increasing $\beta$ increases overdispersal.

#### Total Population Size

In Fig. 3a, there are four different outcomes of climate warming. Working from left to right as we increase *R*, these are as follows. In (i), the population goes extinct for all $p\in [0,1]$. In (ii), $0=c^*_{p=1}<c^*_{p\ne 1}$: fully dispersing populations go extinct for all *c*, while partially sedentary populations can persist for some climate speeds. In (iii), $0\ne c^*_{p=1}<c^*_{p\ne 1}$: there are climate speeds for which the partially sedentary population persists but the fully dispersing population cannot. Finally, in (iv), $c^*_{p=1}>c^*_{p\ne 1}$: the fully dispersing population can persist for higher values of *c* compared to a partially sedentary population. Outcomes (ii) and (iii) correspond to partially sedentary populations performing better than fully dispersing ones. In Fig. 4a, we illustrate how mean dispersal distance ($\beta$) and intrinsic growth rate (*R*) determine which outcome occurs. Black corresponds to outcome (i), white to outcome (iv), and dark grey and light grey corresponds to (ii) and (iii) respectively.

If we are principally interested in how climate shifts affect populations that can always survive in the absence of climate warming, and where partially sedentary populations perform better, one might argue that the light grey region of Fig. 4a is most relevant to our study, the light grey region in Fig. 4a is not large, so we could conclude that partially sedentary populations rarely do better than fully dispersing populations. However, plotting the total population density of the travelling pulse solution as a function of the climate speed, *c*, (Fig. 4b, c) reveals two results. Firstly, for a partially sedentary population, the total population density increases as *c* decreases. Secondly, the total size of the partially sedentary population is almost always greater than that of the fully dispersing population. Both of these features hold in the dark grey, light grey, and white regions of Fig. 4a. So even when a fully dispersing population has a higher critical climate speed (white region, Fig. 4a), the partially sedentary population generally has a larger total population density when the populations persist. The only exception to this is for climate speeds very close to $c^*_{p\ne 1}$. We conclude that partially sedentary populations, with $p\ne 1$, can generally perform better than populations with $p=1$, either by surviving in faster moving climates (dark and light grey region, Fig. 4a), or by having higher population density, or both.

### Shape of the Travelling Pulse Solution and Stability Curves

A notable feature of the stability curves in Fig. 3 are ‘kinks’ which occur at $c=L/N$, for $N=1,2,3,4,\dots$. These ‘kinks’ are due to the stepped nature of the travelling pulse solution shown in Fig. 2. The travelling pulse solution for climate speed *c* has steps in population density at a distance *c*, 2*c*, 3*c*, etc behind the travelling pulse front. In the special case of the top-hat dispersal kernel, we can explicitly construct the travelling pulse solution of the linearised Eq. (7). We demonstrate the role of the non-dispersing fraction in generating these steps in population density, and the corresponding ‘kinks’ in the stability curves. Our result is summarised in Lemma 1.

#### Lemma 1

Let $k(x-y)$ be the top-hat dispersal kernel, $k_T(x-y)$ (3), and let $x,y\in [-L/2,L/2]$. Assume $2\beta >L$ and $c<2\beta -L$. Write $n(x)=\min \{n\ge 1, n\in \mathbb {N}: x+nc\notin [-L/2,L/2]\}$ then the solution of the eigenvalue problem (8) is given by

9 $$\begin{aligned} u(x)=\frac{B}{\lambda ^{n(x)}}\frac{\lambda ^{n(x)}-(R(1-p))^{n(x)}}{\lambda -R(1-p)}, \end{aligned}$$

where

10 $$\begin{aligned} B=\frac{Rp}{4\beta }\int _{-L/2}^{L/2} u(y)\,\mathrm{d}y. \end{aligned}$$

The critical wave speed, $c^*$, satisfies the transcendental equation

11 $$\begin{aligned} c=\frac{\frac{4\beta }{pR}(1-R(1-p)) +(R(1-p))^{N+1}\epsilon -\epsilon }{N-\frac{(R(1-p))^{N+1}-1}{R(1-p)-1}}, \end{aligned}$$

where $$N+1=\lceil L/c\rceil =\max _{x\in [-L/2,L/2]} n(x)$$ and $$\epsilon =L/c-N$$.

#### Proof

The assumption that $2\beta >L$ ensures that the radius of dispersal is larger than the habitat size, and so all dispersing individuals have a chance of leaving the habitat patch. By also requiring a small climate speed ($c<2\beta -L$), it guarantees that $|x+c-y|<2\beta$ and ensures that the dispersal kernel is positive over the habitat patch. The conditions for Jentzch’s theorem are therefore satisfied (Zhou and Kot 2013), and a positive eigenvalue of largest modulus exists, and the eigenvalue problem (8) can be reduced to

12 $$\begin{aligned} \lambda u(x)={\left\{ \begin{array}{ll} B+R(1-p)u(x+c),\quad x\in \left[ -\frac{L}{2},\frac{L}{2}\right] ,\\ 0,\quad \text {otherwise}.\\ \end{array}\right. } \end{aligned}$$

It is straightforward to show that (9) is a solution of (12). In Appendix A, we outline how the eigenfunction (9) is constructed.

The critical wave speed, $c^*$, is found by setting $\lambda =1$ in (9) and substituting this into (10) and using the definition of $\epsilon$ we rearrange to find the expression for *c*. $\square$

The lemma says that if $\lceil L/c\rceil =N+1$, then we expect the travelling pulse solution to have $N+1$ steps in population density (e.g. in Fig. 2, where $L=1$, $c=0.4$ we have $\lceil L/c\rceil =3$, corresponding to the 3 steps in the solution). As we can see from Eq. (9), working backwards from the pulse front, the non-dispersing fraction of the population in the first step have been in this location for 1 year, because the location has just become suitable habitat. In the second step behind the pulse front, the population has been there for 2 years, and the location became suitable habitat 2 years ago, and so on until the $(N+1)$th step, where the non-dispersing population has been at this location for $N+1$ years. These extra years of population growth, that occur towards the trailing edge of the travelling pulse, produce the high population density associated with a partially sedentary population (Fig. 2). The transcendental equation defining the critical wave speed (11) has sharp transitions at $c=L/N$, consistent with Fig. 3. These transitions occur because the integral of *u*(*x*) has a large change in value every time a new step in the travelling pulse solution is introduced. A new step is introduced when *c* is decreased sufficiently, or when *L* is increased sufficiently, to change the value of $\lceil L/c\rceil$.

### Density-Dependent Dispersal, $p=p(n)$

In this section, we let the dispersing fraction be determined by the local density just prior to the dispersal phase. We follow Lutscher (2008) in our choice of the function *p*(*n*) and let $p=g(af(n))$. We focus primarily on positive density-dependence, the most common form of density-dependence (Matthysen 2005), where the fraction dispersing increases with the local population density, as would be observed when competition for resources promotes emigration. The parameter *a* refers to the *sensitivity of dispersal* to local density as described by Lutscher (2008). Small *a* indicates a low sensitivity to density and a propensity to not move. The function *g* is given by

13 $$\begin{aligned} g(u)=1-e^{-u}, \end{aligned}$$

so individuals are more likely to move when the population density is high. Lutscher (2008) discusses other choices of *g* and *p*, including one in which *p* depends on the density at the beginning of the season ($p=g(n)$). We choose Eq. (13) to enable us to directly relate our findings to the work of Lutscher (2008).

Explicitly writing the positive density-dependent dispersal form of Eq. (4) yields

14 $$\begin{aligned}&n_{t+1}(x)=\underbrace{\int ^{L/2+ct}_{-L/2+ct}(1-\exp (-af(n_t(y))))f(n_t(y))k(x-y)\,\mathrm{d}y}_{\text {\tiny dispersing fraction}}\nonumber \\&+\underbrace{\exp (-af(n_t(x)))f(n_t(x))}_{{\mathop {\text {\tiny fraction}}\limits ^{\text {\tiny {non-dispersing}}}}}, \end{aligned}$$

for $x\in \left[ -\frac{L}{2}+c(t+1),\frac{L}{2}+c(t+1)\right]$, and $n_{t+1}(x)=0$ outside of this region. The special case of Eq. (14) with $c=0$ was studied by Lutscher (2008). We cannot conduct a linear stability analysis of Eq. (14) because the choice of density-dependence generates an Allee effect (see (Lutscher 2008)), so instead we investigate the critical climate speed numerically.

**Fig. 5:**
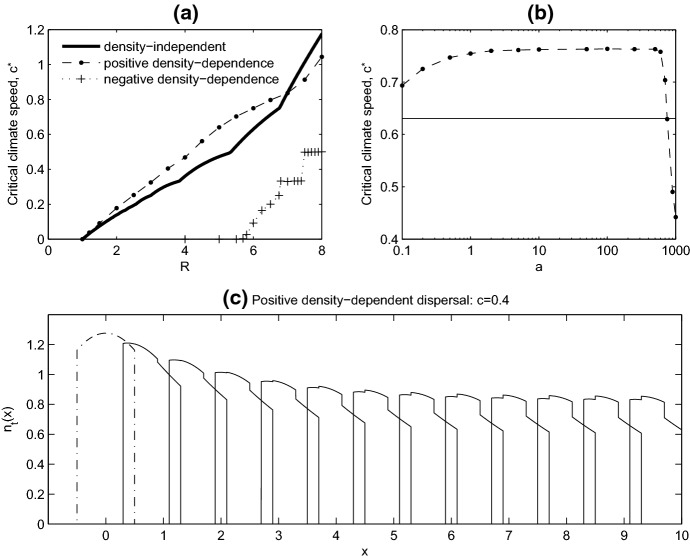
**a** Critical wave speed ($c^*$) plotted as a function of intrinsic growth rate (*R*). We compare $\max c^*_{p\in [0,1]}$ for density-independent dispersal (solid line) to $c^*$ for positive density-dependent dispersal (sensitivity parameter $a=1$, dash-dot line), and to $c^*$ for negative density-dependent dispersal (sensitivity parameter $a=0.1$, crosses). **b** Critical wave speed ($c^*$) is plotted as a function of the dispersal sensitivity parameter, *a*, in the case of positive density-dependent dispersal, with *R*=6. The solid line shows the density-independent maximum ($\max c^*_{p\in [0,1]}$) for comparison. In **a** and **b**, below the curves the population can persist and above the curve the population goes extinct. **c** a plot of the travelling pulse solution in the case of positive density-dependent dispersal, with $c=0.4$, $a=1$ and $R=6$. The solution is plotted every 2 years, and the dashed line indicates the initial condition

Figure 5a illustrates that positive density-dependent dispersal can outperform a population using density-independent dispersal. By this we mean that when *R* is small to intermediate, a population using positive density-dependent dispersal can persist for climate speeds for which a population using density-independent dispersal (any *p*) goes extinct (compare the dash-dot line to the solid line in Fig. 5a). The solid line in Fig. 5a illustrates the highest value of $c^*$ achieved by density-independent dispersal, i.e. $\max _{p\in [0,1]} c^*_{p}$, and the line lies below the dash-dot line, which corresponds to $c^*$ for positive density-dependent dispersal. Figure 5b shows that the critical climate speed for density-dependent dispersal is higher than $\max _{p\in [0,1]} c^*_{p}$ for a wide range of choices of the dispersal sensitivity parameter, *a*. (Note that the plot is on a logarithmic scale for *a*.) Only when *a* is near 1000, very insensitive to local density, does density-dependent dispersal have a lower critical climate speed than the density-independent case.

The high $c^*$ associated with positive density-dependent dispersal can be understood by looking at how this dispersal mechanism affects the travelling pulse solutions. Comparing the travelling pulse solution from density-independent dispersal (Fig. 2a) to the corresponding one for density-dependent dispersal (Fig. 5c), we see that density-dependent dispersal lowers the density at the rear of the travelling pulse. As we increase the speed of climate warming (*c*), the population at the rear of the travelling pulse would be the first to be lost due to climate warming, and so density-dependent dispersal instead insures these individuals preferentially move, leading to the higher value of $c^*$ associated with density-dependent dispersal. The higher value of $c^*$ does come at a cost, because overall population density is lower for density-dependent dispersal compared to density-independent dispersal as illustrated by the dots in Fig. 4a, b.

For completeness, we also considered negative density-dependent dispersal by choosing $g(u)=e^{-u}$, individuals have a higher propensity to disperse when density is low, as might associated with group behaviour. When $a=1$ the population went extinct for all $1<R<8$, regardless of the value of *c*, and a much higher intrinsic growth rate was needed for persistence. Decreasing *a* to 0.1 (corresponding to a high sensitivity to population density), we find the population can persist for low *c*, but performs poorly compared to a population using positive density-dependent or density-independent dispersal (Fig. 5a crosses). By only moving when density is low, negative density-dependent dispersal puts the population at higher risk of extinction.

## Discussion

The Fifth Assessment Report of the Intergovernmental Panel on Climate Change (IPCC) found an average increase in global surface temperatures of 0.72 $^\circ$C over the period 1951–2012  (Stocker et al. 2013). Among the many ways in which climatic conditions affect species, temperature often shapes geographical range limits. Increases in temperature shift the location of a species’ climatically suitable range either northwards or up gradients in elevation, and many species have shifted their geographic range in response to the shift in their climate niche (Walther et al. 2002; Parmesan and Yohe 2003). For a species to shift its geographic range requires dispersal, and this naturally leads to the question asked by Zhou and Kot (2011) and others, “can a species keep pace with climate change?” If a species fails to keep pace with climate change or adapt in other ways, then it risks extinction. Most mechanistic models addressing this question (except Harsch et al. (2014)) have assumed the entire population is able to disperse (Santini et al. 2016), when in fact partially sedentary populations, where only a fraction of the population disperse each year, are very common in nature. One might expect that if only a fraction of the population disperses then the population would struggle to keep pace with climate change compared to a population where all individuals disperse each year. In fact, we find that this is not always the case.

We found that partially sedentary populations can outperform fully dispersing populations in two ways: (i) for moderate intrinsic growth rates, partially sedentary populations can persist and track changing climate conditions that a fully dispersing population cannot, and (ii) when both populations persist, the partially sedentary population has the higher density. These two results also hold when dispersal is density-dependent, with the probability of dispersing increasing with local population density. Our work highlights the potentially important role that non-dispersing individuals can play in the survival of range shifting populations.

By constructing a moving-habitat IDE model of a partially sedentary population, building on the work of Zhou and Kot (2011) and Lutscher (2008), we calculated the critical climate speed, $c^*$, and its dependence on the fraction of dispersers in the population, *p*. At climate speeds below $c^*$, the population can persist, and at speeds above $c^*$, the population becomes extinct. One of our main findings is that for $p<1$, $c^*$ can be higher than the critical speed found by Zhou and Kot (2011), who assumed a fully dispersing population. The non-dispersing fraction of the population acts to counter the population loss caused by overdispersal of the dispersing fraction. Partially sedentary populations are then able to persist at climate speeds that a fully dispersing population cannot (Fig. 3). Overdispersal has already been shown to have a role in reducing $c^*$ when mean dispersal distance is increased above a threshold value (Zhou and Kot 2011), and our results show that the non-dispersing fraction of a population can counteract these effects.

Moving-habitat models that include behavioural responses to habitat boundaries can produce values of $c^*$ that are higher than those found by Zhou and Kot (2011). Species that prefer to remain in the suitable habitat, particularly where “preference at the trailing edge is much more important than at the leading edge” (MacDonald and Lutscher 2018), can tolerate higher climate speeds. The non-dispersing fraction in our moving-habitat model essentially show preference to remain in the suitable habitat, so in this regard, our results are consistent with those of MacDonald and Lutscher (2018). Moreover, in our model, the non-dispersers at the trailing edge have spent longer in the habitat than those closer to the leading edge, agreeing with the finding of MacDonald and Lutscher (2018) that the trailing edge can drive increases in $c^*$.

Discrete steps in density occur in the travelling pulse solutions of Eq. 4. The steps occur at distances of $c, 2c, 3c, \ldots$ back from the leading edge of the pulse, *c* being the velocity of climate change. These steps are caused by the non-dispersing fraction of the population, who have spent, respectively, 1 year, 2 years, 3 years, ... in the suitable habitat. These steps can make the population sensitive to changes in habitat size, *L*. For example, consider Fig. 2a, where the climate velocity is $c=0.4$. Reducing *L* from 1 to 0.8 results in $\lceil L/c\rceil$ changing from 3 to 2, and the population goes from having three steps in population density to two. The change in number of steps leads to a sharp drop in total population density. In contrast, if a population experiences a sufficiently large increase in *L*, we get a sharp increase in total density. Such increases or decreases in *L* could result from anthropogenic change, or from changes in biotic interactions as we discuss later.

Comparing density-dependent dispersal to density-independent dispersal, we found that positive density-dependence allowed a population to survive at higher climate speeds, speeds at which a density-independent dispersing population would go extinct. For a stationary habitat ($c=0$), increasing sensitivity of dispersal to local density increases the speed of travelling wave solutions of the IDE and decreases the critical habitat size required for population persistence (Lutscher 2008). The speed of these travelling wave solutions is relevant here, as it provides an upper bound on the maximum speed of climate shift that can be tolerated by a density-dependent population and explains the higher values of $c^*$ associated with positive density-dependent dispersal in our model (Fig. 5a). Figure 5a also shows that population density is much lower for positive density-dependent dispersing populations, because overdispersal, and hence population loss, is higher than in a density-independent partially sedentary population.

While much evidence shows that a species’ range limits are constrained by climate, biotic factors such as competition and predation can also influence the range limits (HilleRisLambers et al. 2013). For example, empirical evidence shows competition can constrain the lower range limit of conifers and mountain hemlock in Mt. Rainier National Park. Although we do not consider these biotic factors in our model, biotic constraints would affect the trailing edge of the travelling pulse solution, where population density is highest in our model. Negative interactions such as interspecific competition or predation at the lower range limit are likely to lower population density of partially sedentary populations as the interactions would preferentially affect the locations of highest population density.

In nature, the speed of a species’ range shift can be faster or slower than the climate-induced shift, which HilleRisLambers et al. (2013) refers to as ‘ecological surprises’. These ecological surprises occur when there are changes to species interactions in the new climatically suitable range. For instance, if new positive interspecific interactions (e.g. facilitative effects) occur in the new range, species can accelerate their range shifts by taking advantage of the improved growth accompanying these interactions. A partially sedentary population would not get the full benefit from these facilitative interactions, as only the dispersing fraction would reach the new climatically suitable locations occupied by the facilitating species. By contrast, if interspecific interactions in the new climatically suitable range are negative (e.g. encounter with a new predator) then we expect a partially sedentary population to suffer less from these negative effects compared to a fully dispersing population.

Our moving-habitat IDE assumes that the intrinsic population growth rate is uniform across the range of the species and drops off sharply at habitat boundaries. We expect that many species would not have such an extreme relationship between growth and habitat suitability. More realistic growth functions, where the intrinsic growth rate depends directly on the temperature at each location in the habitat, result in a situation where intrinsic growth is highest in the interior of the species range and declines gradually towards range boundaries (Hurford et al. 2019). Fully dispersing populations with right-skewed growth functions (long right tail), have been shown to exhibit lower population abundance, and lag behind climate change when compared to populations with left-skewed growth functions (long left tail) (Hurford et al. 2019). The travelling pulse solutions associated with partially sedentary populations in our model are right-skewed, but have higher population densities than a corresponding fully dispersing population. Introducing more realistic choices of growth function into our model may act to reverse some of our findings, a right-skewed growth function may lead to population loss that counteracts the population increases associated with a partially sedentary population, but the precise strength of interaction between growth curve skew and population density skew is unclear and the topic of future research.

We do not consider habitat connectivity in our study, and instead assume the newly suitable habitat is completely accessible to the species. In practice, the new climatically suitable range might include habitat unsuitable for growth, even though the climate conditions are suitable (Littlefield et al. 2019). There is an extensive body of work exploring the role of habitat heterogeneity in determining travelling wave speeds in IDE models (Dewhirst and Lutscher 2009; Crone et al. 2019), and combining this with the partially sedentary moving-habitat model presented here would be a natural step towards formulating a more realistic description of range shifting populations faced with the challenge of poor landscape connectivity.

We have shown that the non-dispersing fraction of a population can play an important role in a species’ ability to keep pace with climate change, by mitigating the effects of overdispersal. In plants, seed bank persistence (resulting in a non-dispersing fraction of the population) has been identified as one of the most important factors in explaining range size under environmental change (Estrada et al. 2015). The lack of studies of climate-driven range shifts that incorporate dispersal and life history traits prevent us from identifying if seed banks are mitigating the effects of overdispersal, as we propose, or simply allowing persistence under unsuitable conditions, or both (Estrada et al. 2015). As more empirical studies become available, it may be possible to test this and other predictions from our study, namely that partially sedentary populations have higher population densities and would be more susceptible to changes in habitat size than their fully dispersing counterparts. Our findings highlight that life history strategies can have counterintuitive effects on survival of species experiencing climate-induced range shifts. The role of non-dispersers should not be overlooked.

## Appendix A

We construct the eigenfunction in Eq. (9). Given $x\in [-L/2,L/2]$, suppose for some $N\in \mathbb {N}$, $n(x)=N+1$ in Lemma 1. So, $x+c, x+2c, x+3c,\dots , x+Nc\in [-L/2,L/2]$, but $x+(N+1)c\notin [-L/2,L/2]$. From Eq. (12), we have

15 $$\begin{aligned} u(x)={\left\{ \begin{array}{ll} \frac{B}{\lambda }+\frac{R(1-p)}{\lambda }u(x+c),\quad x\in \left[ -\frac{L}{2},\frac{L}{2}\right] ,\\ 0,\quad \text {otherwise}.\\ \end{array}\right. } \end{aligned}$$

Treating (15) as a recursion relation relating *u*(*x*) to $u(x+c)$, we iterate repeatedly to obtain:

16

Since $x+(N+1)c\notin [-L/2,L/2]$, we have $u(x+(N+1)c)=0$ and

17 $$\begin{aligned} u(x)=\frac{B}{\lambda }\left[ \sum _{i=0}^N\left( \frac{R(1-p)}{\lambda }\right) ^i\right] =\frac{B}{\lambda ^{N+1}}\frac{\lambda ^{N+1}-(R(1-p))^{N+1}}{\lambda -R(1-p)}, \end{aligned}$$

as required.
